# Supplementary material for: Synergy of multiple precipitate/matrix interface structures for a heat resistant high-strength Al alloy
Source: Nat Commun. 2023 May 23;14:2959. doi: 10.1038/s41467-023-38730-z (PMC10205818; doi:10.1038/s41467-023-38730-z)
Supplement: Supplementary file 1 — Supplementary Information [file 41467_2023_38730_MOESM1_ESM.pdf]

Supplementary Information

Synergy of multiple precipitate/matrix interface structures for a heat resistant high-strength Al alloy

Qiang Lu, Jianchuan Wang, Hongcheng Li, Shenbao Jin, Gang Sha, Jiangbo Lu, Li Wang, Bo Jin, Xinyue Lan, Liya Li, Kai Li*, Yong Du *

*Corresponding authors: leking@csu.edu.cn (K. Li); yong-du@csu.edu.cn (Y. Du)

This file includes:

Supplementary Figure 1. 3DAP results of the designed alloy under peak ageing.

Supplementary Figure 2. The mechanical properties of the Al-4Cu alloy.

Supplementary Figure 3. Changes in the size of the θ′-Al_2Cu precipitates in the peak-aged Al-4Cu alloy before and after thermal exposure heat treatment.

Supplementary Figure 4. Atomic resolution EDX elemental maps of χ-AgMg interface phase on different θ′ precipitates.

Supplementary Figure 5. The EDS results of different χ-AgMg interface phases.

Supplementary Figure 6. The proxigram of total χ-AgMg interface phase (at all interfaces) marked in pink.

Supplementary Figure 7. Different AgMg or Ag segregation structures.

Supplementary Figure 8. HAADF-STEM images and EDX results of precipitates after thermal exposure at 210°C for 100 h.

Supplementary Figure 9. 3DAP results of the designed alloy in the underaged state.

Supplementary Figure 10. HAADF-STEM images and EDX maps of an independently precipitated L phase.

Supplementary Figure 11. CALPHAD results of the Al-Cu(-Sc-Ag-Mg-Si) alloys under different conditions.

Supplementary Figure 12. The micro-scale constituents of the designed alloy.

Supplementary Figure 13. The radial distribution function (RDF) of pure aluminum at different temperatures.

Supplementary Figure 14. The ab initio molecular dynamics (AIMD) results of pure Al calculated at different temperatures.

Supplementary Figure 15. The stress-strain curves of the designed alloy under different ageing states.

Supplementary Table 1. The mechanical properties of Al alloys before and after thermal exposure

Supplementary Note 1. The mechanical properties and microstructure of Al-4Cu alloy

Supplementary Note 2. The micro-scale constituents of the designed alloy in different states

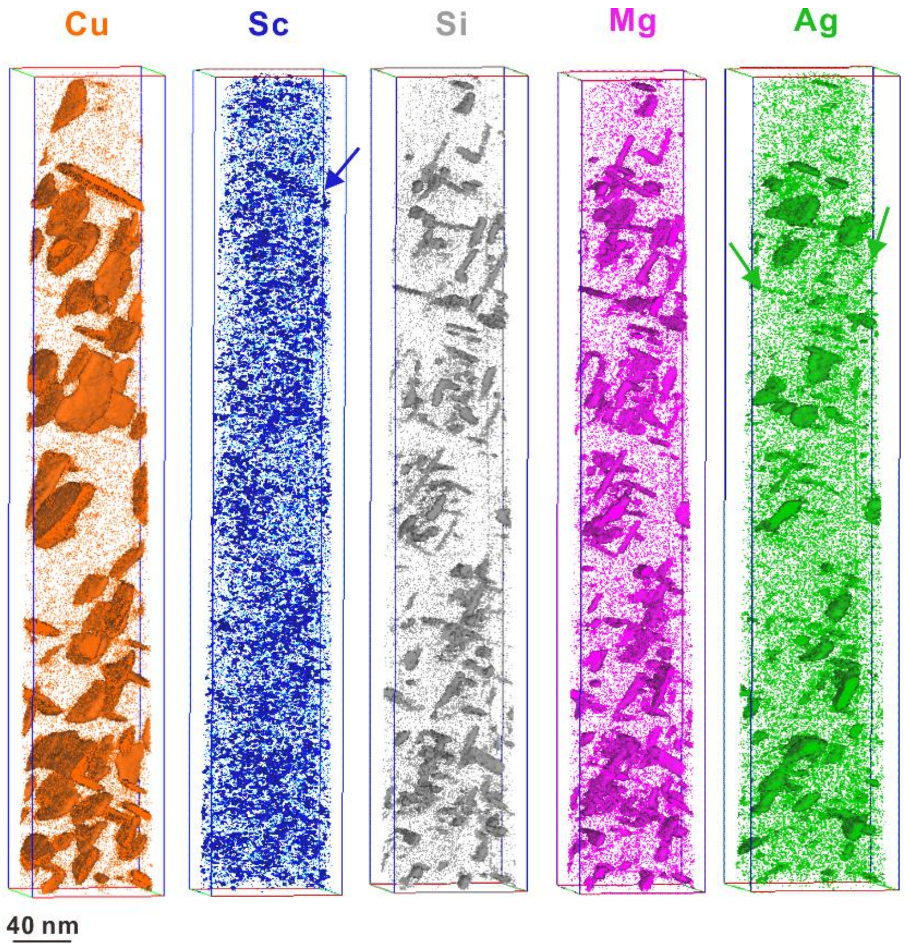

Supplementary Figure 1. **3DAP results of the designed alloy under peak ageing.** The iso-surface of Cu, Sc, Si, Mg and Ag are set as 9.0 at.%, 0.3 at.%, 2.0 at.%, 3.0 at.% and 1.0 at.%, respectively.

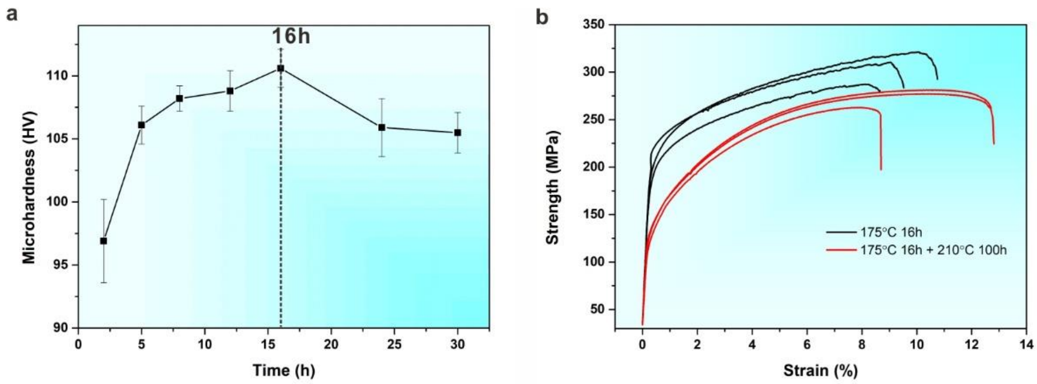

Supplementary Figure 2. **The mechanical properties of the Al-4Cu alloy. a** Microhardness of the Al-4Cu alloy aged for different times at 175℃. **b** Stress-strain curves of the peak-aged Al-4Cu alloy before and after thermal exposure. The error bars denote the standard deviation of the mean.

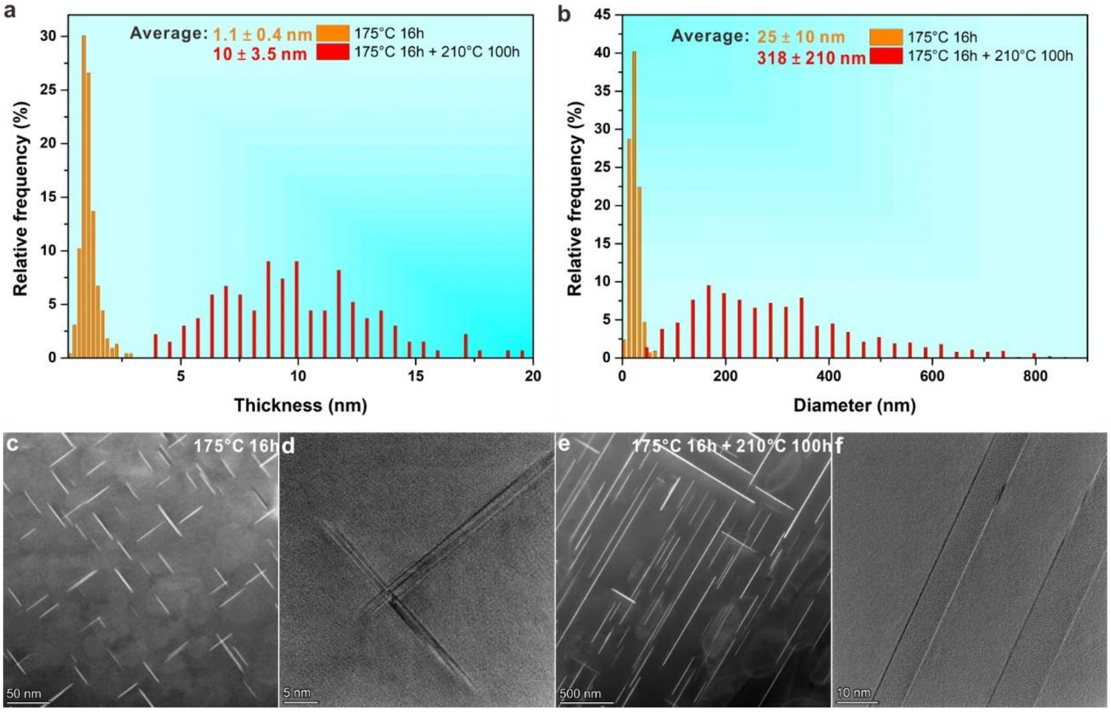

Supplementary Figure 3. **Changes in the size of the θ′-Al_2Cu precipitates in the peak-aged Al-4Cu alloy before and after thermal exposure heat treatment. a** and **b** Distributions of thickness and diameter of the Al-4Cu alloy, for both the peak-aged and thermally exposed states. **c** and **d** Typical images used to measure the diameter and thickness of θ′-Al_2Cu precipitates in the peak-aged state of the current alloy. **e** and **f** Typical images for the state after thermal exposure.

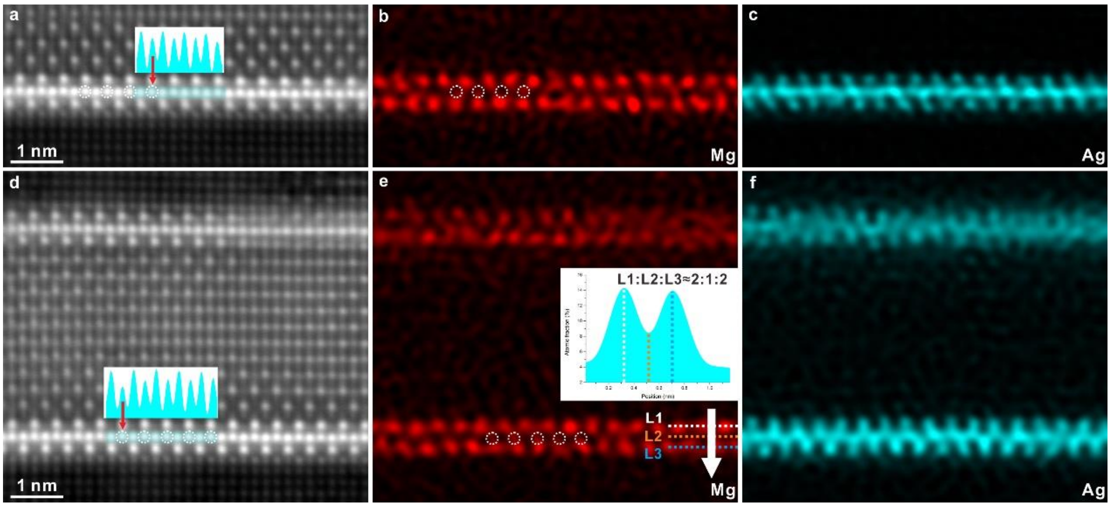

Supplementary Figure 4. **Atomic resolution EDX elemental maps of χ-AgMg interface phase on different θ′ precipitates. a** Atomic resolution HAADF-STEM image of a θ′ precipitate with χ-AgMg interface phase only on one side. **b** and **c** Atomic resolution EDX elemental maps of the area shown in (**a**). **d** Atomic resolution HAADF-STEM image of a θ′ precipitate with χ-AgMg interface phase on both sides. **e-f** Atomic resolution EDX elemental maps of the area shown in (**d**). The atomic columns marked with white circles in (**b**) and (**e**) correspond to those similarly marked in (**a**) and (**d**), respectively.

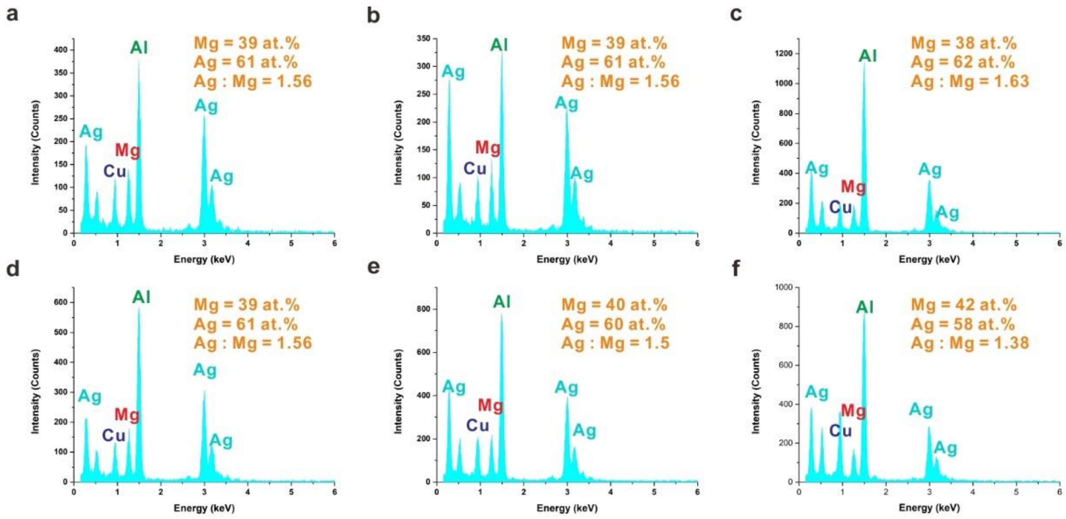

Supplementary Figure 5. **The EDS results of different χ-AgMg interface phases. a-f** EDX spectra of different χ-AgMg phase particles. The average content of Ag in the χ-AgMg phase is 61 ± 1 at.%, while that of Mg is 39 ± 1 at.%. The average atomic ratio of Ag to Mg in the χ-AgMg interface phase is about 1.56.

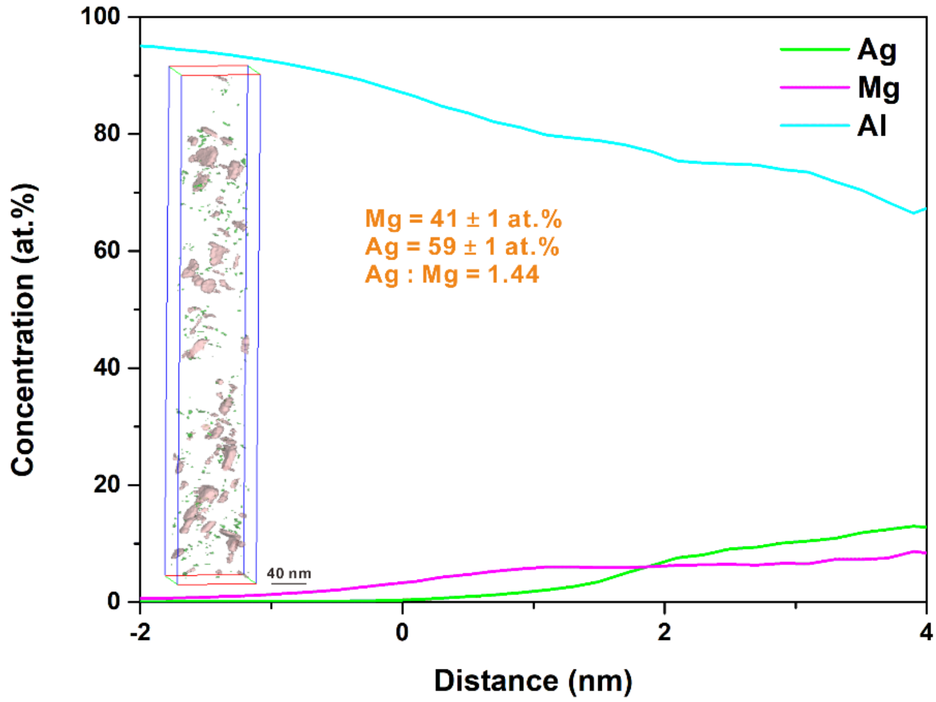

Supplementary Figure 6. **The proxigram of total χ-AgMg interface phase (at all interfaces) marked in pink.** The atomic ratio of χ-AgMg interface phase is about 1.44.

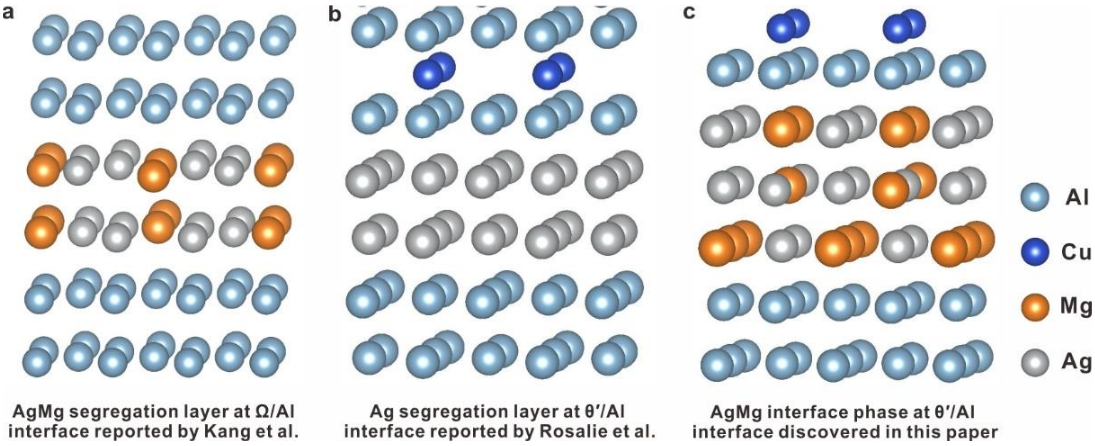

Supplementary Figure 7. **Different AgMg or Ag segregation structures. a** Structure of the uniformly distributed AgMg segregation layer containing double atomic layers at the Ω/Al interface reported by Kang et al.[1, 2]. **b** Structure of the Ag segregation layer with double atomic layers at the θ′/Al interface reported by Rosalie et al.[3]. **c** Structure of the newly discovered χ-AgMg interface phase at the θ′/Al interface in this work.

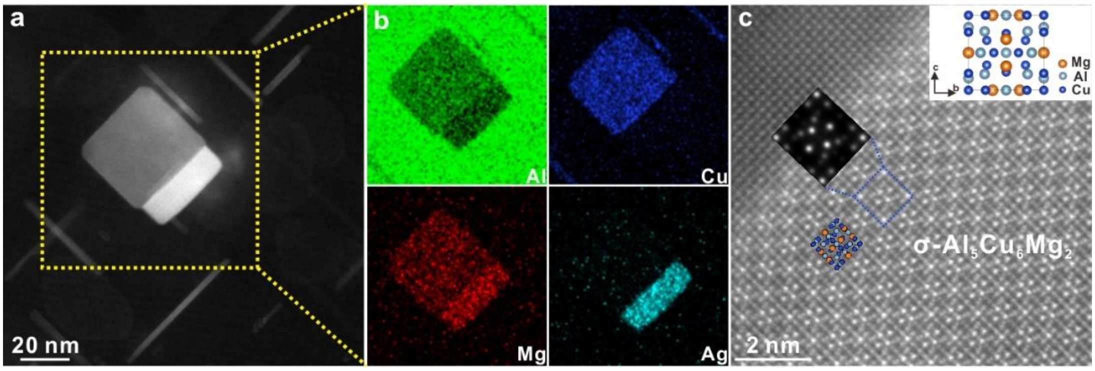

Supplementary Figure 8. **HAADF-STEM images and EDX results of precipitates after thermal exposure at 210℃ for 100 h. a** Low magnification HAADF-STEM image. **b** EDX elemental maps of σ and the thick AgMg-rich ξ phase. **c** atomic resolution HAADF-STEM of σ-$Al_5Cu_6Mg_2$ precipitate. The HAADF-STEM image simulated by QSTEM is inserted in (**c**).

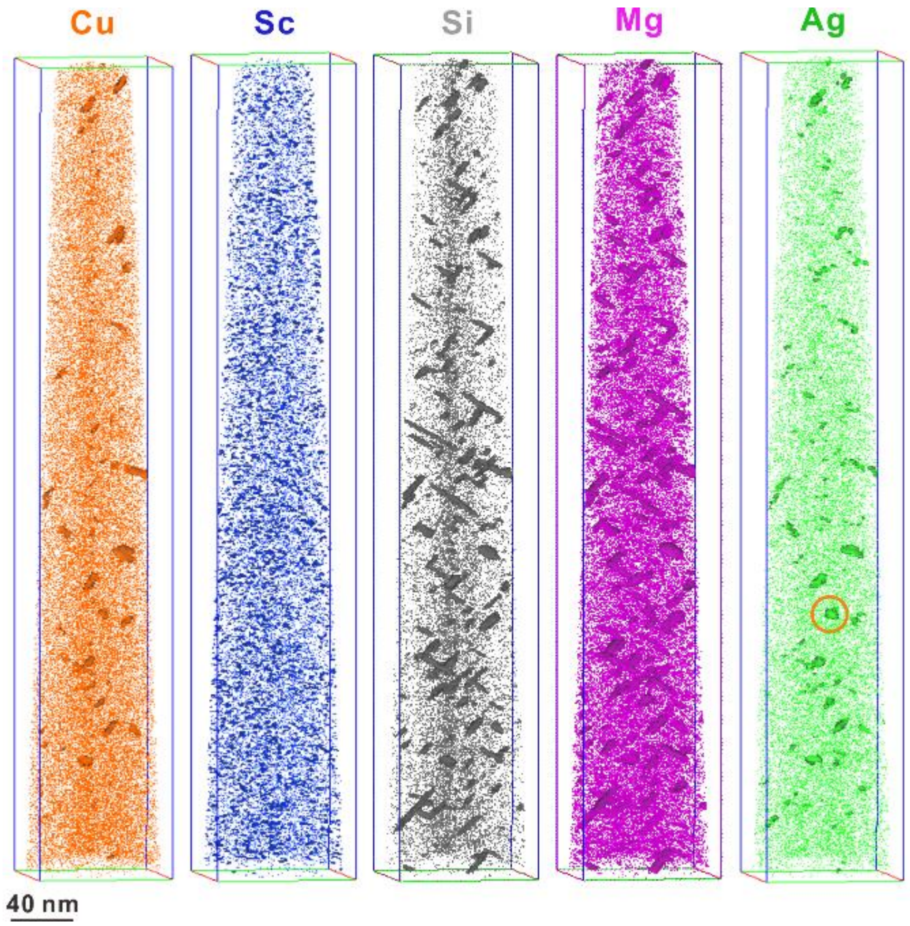

Supplementary Figure 9. **3DAP results of the designed alloy in the underaged state.** The iso-surface of Cu, Sc, Si, Mg and Ag are set as 9.0 at.%, 0.3 at.%, 2.0 at.%, 3.0 at.% and 1.0 at.%, respectively.

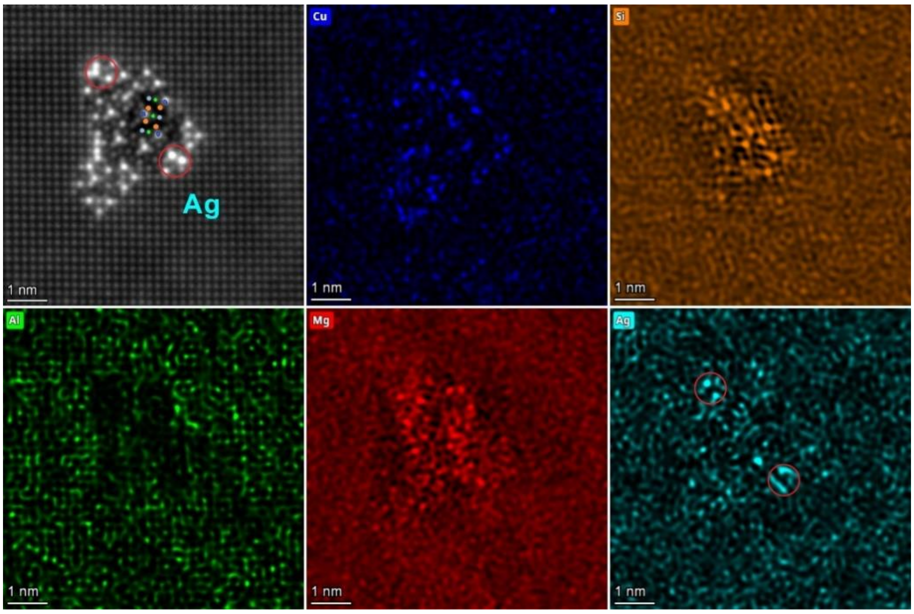

Supplementary Figure 10. **HAADF-STEM images and EDX maps of an independently precipitated L phase.**

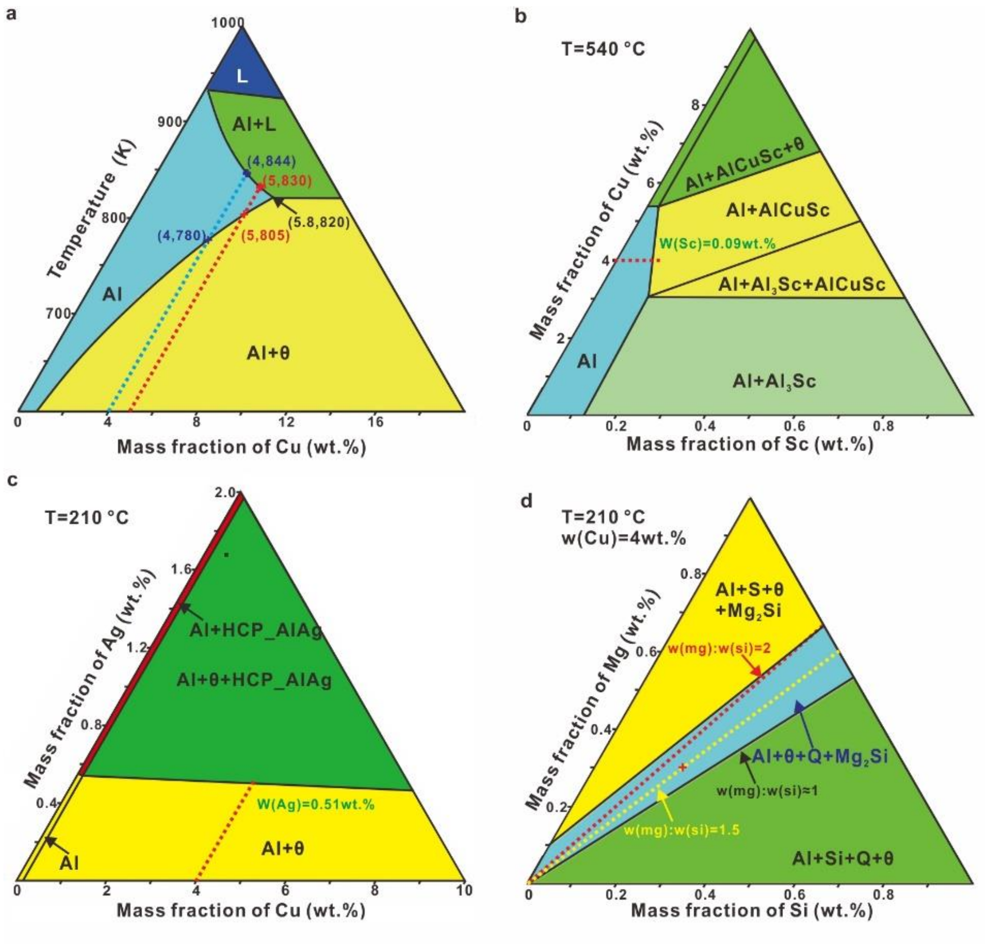

Supplementary Figure 11. **CALPHAD results of the Al-Cu(-Sc-Ag-Mg-Si) alloys under different conditions. a** Vertical section phase diagram of the Al-Cu binary alloy. **b** Ternary isothermal section of Al-Cu-Sc system at 540°C. **c** Ternary isothermal sections of Al-Cu-Ag systems at 210°C**. d** Isothermal sections of Al-Mg-Si(-4Cu) systems at 210°C.

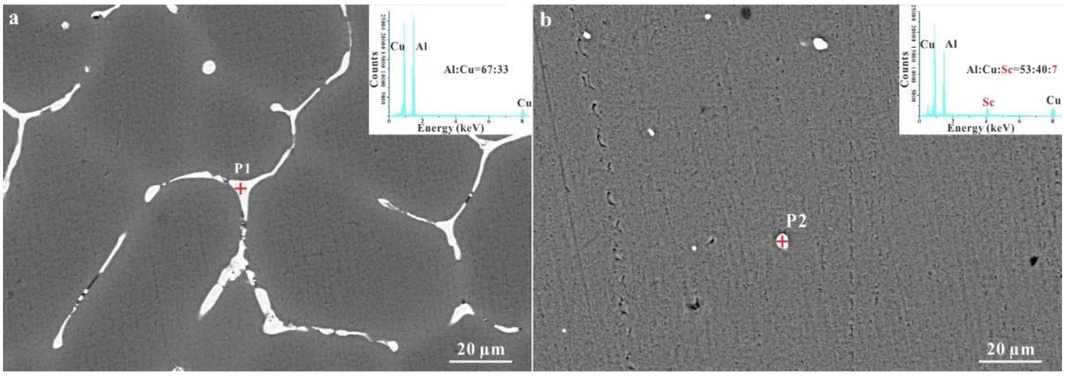

Supplementary Figure 12. **The micro-scale constituents of the designed alloy. a** Micro-scale constituent in as-cast state. **b** Micro-scale constituents after homogenization. The EDX Spectrums of P1 and P2 are inserted in the upper right corner of (**a**) and (**b**), respectively.

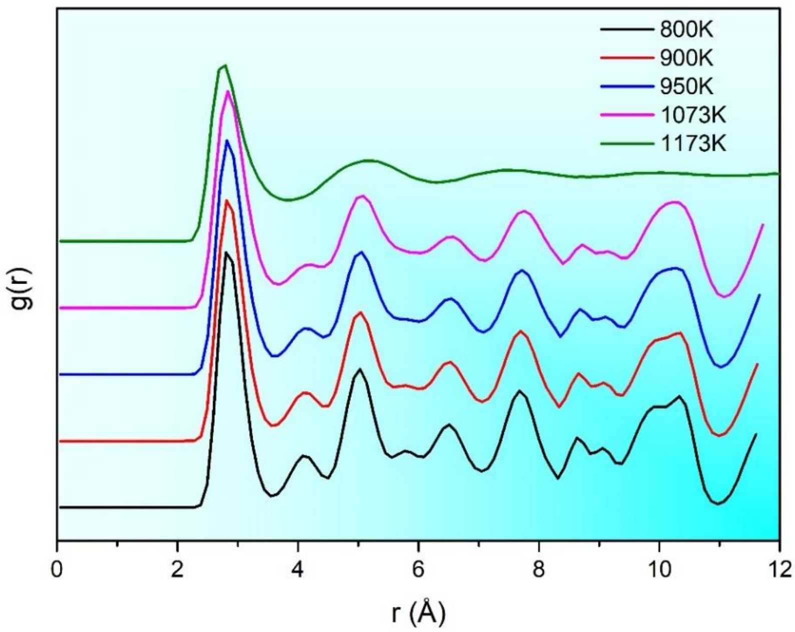

Supplementary Figure 13. **The radial distribution function (RDF) of pure aluminum at different temperatures.**

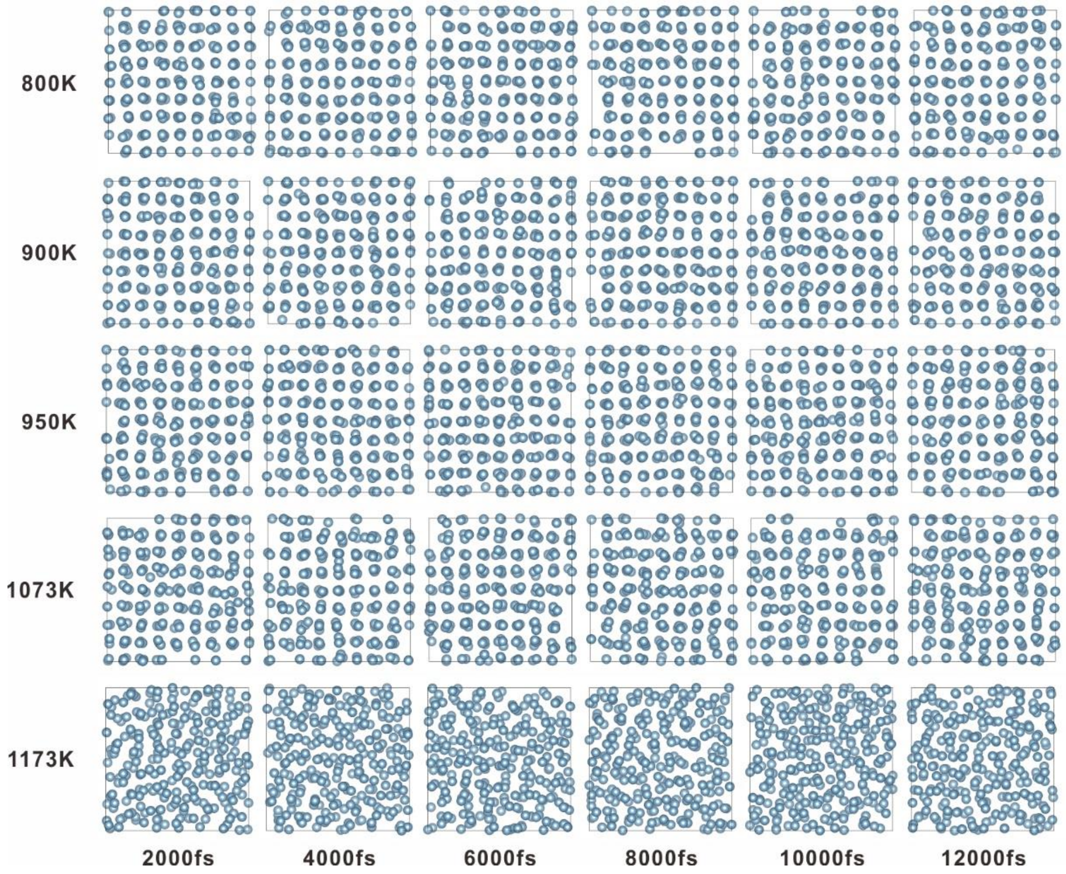

Supplementary Figure 14. **The ab initio molecular dynamics (AIMD) results of pure Al calculated at different temperatures.**

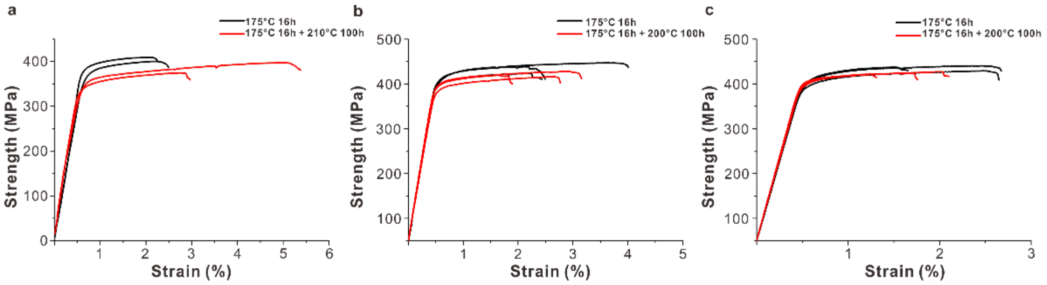

Supplementary Figure 15. **The stress-strain curves of the designed alloy under different ageing states.** **a** Stress-strain curves of the peak-aged Al-4.15Cu-0.3Mg-0.47Ag-0.17Si-0.1Sc alloy before and after thermal exposure at 210 ℃. **b** and **c** Stress-strain curves of the peak-aged Al-3.90Cu-0.3Mg-0.45Ag-0.18Si-0.09Sc and Al-3.86Cu-0.3Mg-0.46Ag-0.17Si-0.09Sc alloys before and after thermal exposure at 200 ℃, respectively.

Supplementary Table 1. The mechanical properties of Al alloys before and after thermal exposure

Composition	YS before (MPa)	YS after (MPa)	Retention ratio (%)	Temperature (℃)	Composition	YS before (MPa)	YS after (MPa)	Retention ratio (%)	Temperature (℃)
2099-T83[4]	502	413	82.3	180	AA3004[5]	104	104	100	300
2397-T87[4]	423	370	87.5	180	Al-1.2Mn-0.75Si-0.75Cu-0.3Mg[6]	179	151	84.4	200
AA2618[7]	369	311	84.3	200	Al-1.2Mn-0.75Si-0.3Mg[6]	99	76	76.8	200
2618-AlN[7]	371	332	89.5	200	Al-1.2Mn-0.75Si-0.5Cu-0.1Mg[6]	64	64	100	200
Al-5.3Cu-0.8Mg-0.5Ag-0.3Mn-0.15Zr[8]	446	226	50.7	200	Al-1.2Mn-0.75Si-0.75Cu[6]	55	53	96.4	200
Al-4.9Cu-0.7Mg-0.36Ag-0.3Mn-0.2Zr[9]	456	312	68.4	200	Al-1.2Mn-0.75Si-0.5Cu[6]	50	49	98	200
Al-4.8Cu-0.8Mg-0.14Ag-0.3Mn-0.2Zr[9]	425	305	71.8	200	Al-6.87Si-0.61Mg-0.1Fe[10]	248	95	38.3	250
Al-6.2Cu-0.6Mg-0.5Ag-0.3Mn-0.2Zr-0.2Er[11]	441	341	77.3	200	Al-6.87Si-0.61Mg-0.1Fe-0.1Zr[10]	249	117	47	250
Al-6.6Cu-0.6Mg-0.5Ag-0.3Mn-0.14Zr[11]	472	394	83.5	200	Al-12Si-4Cu-1Mg-2Ni[12]		219	88.4	200
Al-4.72Cu-0.45Mg-0.54Ag-0.17Zr[13]	414	349	84.3	200	Al-12Si-4Cu-1Mg-2Ni[12]		128	68.6	250
Al-5.5Cu-1.0Mg-1.2Ag[14]	481	323	67.2	200	Al-8.5Si-1.76Cu-0.55Mg-0.32Zr[15]	323	162	49.7	250
Al-5.3Cu-0.8Mg-0.6Ag-0.4Mn-0.1Zr[16]	482	300	62.2	200	Al-8.4Si-1.7Cu-0.6Mg-0.33Zr-1.9Ni[15]	354	185	52.4	250
Al-5.3Cu-0.8Mg-0.6Ag-0.4Mn-0.1Zr-0.45Ce[16]	528	345	65.3	200	Al-8.6Si-1.8Cu-0.55Mg-0.33Zr-0.75Mn[15]	356	169	47.5	250
7085-T7651[4]	468	258	55.1	180	Al-4Cu-0.5Mg-0.5Ag-0.19Si-0.09Sc (this work)	405	289	71.4	210
7085-T7452[4]	497	267	53.7	180	Al-4Cu-0.5Mg-0.5Ag (this work)	387	227	58.7	210
7075-T7651[4]	460	237	51.5	180	Al-4Cu-0.28Mg-0.4Ag (this work)	341	225	65.9	210
6082[17]	341	322	94.4	150	Al-4Cu-0.28Mg-0.6Ag (this work)	352	250	71	210
6082[17]	341	239	70.1	175	Al-4Cu-0.8Mg-0.8Ag-0.2Si-0.1Sc (this work)	424	238	56.1	210
Al-Mg-Si[18]	280 (UTS)		81	200	Al-4Cu-0.8Mg-0.8Ag-0.3Mn-0.15Zr (this work)	446	250	56.1	210
Al-Mg-Si-0.1La[18]	290 (UTS)		81.6	200	Al-4Cu-0.315Mg-0.5Ag-0.21Si-0.09Sc (The designed alloy)	383	346	90.3	210
Al-Mg-Si-0.22La[18]	265 (UTS)		92	200	Al-4Cu-0.315Mg-0.5Ag-0.21Si-0.09Sc (The designed alloy)	413	401	97.1	200
Al-Mg-Si-0.32La[18]	240 (UTS)		92	200					

Supplementary Note 1. The mechanical properties and microstructure of Al-4Cu alloy

To visualize the inhibitory effect of multiple segregation structures on the coarsening of the precipitates, a control alloy without multiple segregation structures was designed. The results of previous studies showed that Ag[3], Sc[19] and Si[20, 21] elements could segregate at the θ'/Al interface, thus forming segregation structures at the precipitate/matrix interface. In addition, the addition of Mg will promote the formation of S'-Al_2CuMg precipitates, resulting in different types of precipitates in the alloy. Therefore, the alloy composition of the control alloy is set as Al-4Cu (in wt.%).

Supplementary Fig. 2 shows the mechanical properties of the control alloy under different conditions. The microhardness results indicate that the Al-4Cu alloy reaches peak aging after aging at 175°C for 16 h. In addition, the yield strength values of the peak-aged Al-4Cu alloy before and after thermal exposure are 208 ± 12 and 137 ± 3 MPa, respectively. The strength retention ratio of the Al-4Cu alloy is 65.9%, which indicates that the strength of the alloy decreases significantly after thermal exposure at 210°C for 100 h. Furthermore, rapid coarsening of the precipitates was observed as shown in Fig. S3. According to Supplementary Figs. 3a-b, the average thickness values of θ′-Al_2Cu before and after thermal exposure are 1.1 ± 0.4 nm and 10 ± 3.5 nm, while those values for diameter are 25 ± 10 nm and 318 ± 210 nm. The statistical results show that θ′-Al_2Cu precipitates without multiple segregation structures have been obviously coarsened after thermal exposure, thereby leading to the rapid decrease in tensile properties of the Al-4Cu alloy. That is, the presence of the multiple segregation structures at the θ′/Al interface is the main cause of effectively inhibiting the coarsening of θ′-Al_2Cu precipitates in the designed Al-4Cu-0.315Mg-0.5Ag-0.21Si-0.09Sc alloy.

Supplementary Note 2. The micro-scale constituents of the designed alloy in different states

According to Supplementary Fig. 12, the main micro-scale constituents in the as-cast alloy are θ-Al_2Cu phase, and the small amount of micro-scale constituents in the homogenized alloy are the dot-shaped AlCuSc phase. After the homogenization heat treatment, most of the constituents in the as-cast alloy were dissolved in the aluminum matrix, which ensures that there are enough supersaturated solutes in the aluminum matrix for the formation of nanoscale precipitates.

However, in the part of thermodynamic calculations, the designed alloy should not contain micron-scale constituents after homogenization heat treatment, which is not fully in agreement with the experimental results. According to the ICP results, the actual composition of the designed alloy is Al-4.15Cu-0.3Mg-0.47Ag-0.17Si-0.1Sc. The content of Sc in the actual composition is higher than the maximum solubility of Sc, which is 0.09 wt.% at 540°C. Therefore, the excess Sc forms the small amount of dot shaped AlCuSc constituents during the homogenization heat treatment.

Supplementary References

1. Kang, S. J., Kim, Y.-W., Kim, M.&Zuo, J.-M. Determination of interfacial atomic structure, misfits and energetics of Ω phase in Al–Cu–Mg–Ag alloy. *Acta Mater.* **81**, 501-511 (2014).

2. Kang, S. J., Zuo, J.-M., Han, H. N.&Kim, M. Ab initio study of growth mechanism of omega precipitates in Al-Cu-Mg-Ag alloy and similar systems. *J. Alloy. Compd.* **737**, 207-212 (2018).

3. Rosalie, J. M.&Bourgeois, L. Silver segregation to θ′ (Al_2Cu)–Al interfaces in Al–Cu–Ag alloys. *Acta Mater.* **60**, 6033-6041 (2012).

4. Jabra, J. et al. The effect of thermal exposure on the mechanical properties of 2099-T6 die forgings, 2099-T83 extrusions, 7075-T7651 plate, 7085-T7452 die forgings, 7085-T7651 plate, and 2397-T87 plate aluminum alloys. *J. Mater. Eng. Perform.* **15**, 601-607 (2006).

5. Liu, K.&Chen, X. G. Development of Al–Mn–Mg 3004 alloy for applications at elevated temperature via dispersoid strengthening. *Mater. Des.* **84**, 340-350 (2015).

6. Shohei, I.&Masami, A. Effect of additional Cu and Mg in Al–Mn–Si alloy on intergranular corrosion susceptibility after heating at 200°C. *J. Jpn. Inst. Light Met.* **59**, 108-113 (2009).

7. Wang, J. H.&Yi, D. Q. Preparation and Properties of Alloy 2618 Reinforced by Submicron AlN Particles. *J. Mater. Eng. Perform.* **15**, 596-600 (2006).

8. Song, Y.-f., Pan, Q.-l., Wang, Y., Li, C.&Feng, L. Elevated-temperature mechanical properties and thermal stability of Al-Cu-Mg-Ag heat-resistant alloy. *J. Cent. South. Univ.* **21**, 3434-3441 (2014).

9. Zhou, X., Liu, Z., Bai, S., Liu, M.&Ying, P. The influence of various Ag additions on the nucleation and thermal stability of Ω phase in Al–Cu–Mg alloys. *Mater. Sci. Eng. A* **564**, 186-191 (2013).

10. Tzeng, Y.-C., Chengn, V.-S., Nieh, J.-K., Bor, H.-Y.&Lee, S.-L. Microstructure and Thermal Stability of A357 Alloy With and Without the Addition of Zr. *J. Mater. Eng. Perform.* **26**, 5511-5518 (2017).

11. Bai, S., Huang, T., Xu, H., Liu, Z., Wang, J.&Yi, X. Effects of small Er addition on the microstructural evolution and strength properties of an Al–Cu–Mg–Ag alloy aged at 200°C. *Mater. Sci. Eng. A* **766**, 138351 (2019).

12. Liu, H., Pang, J., Wang, M., Li, S.&Zhang, Z. The effect of thermal exposure on the microstructure and mechanical properties of multiphase AlSi12Cu4MgNi2 alloy. *Mater. Charact.* **159**, 110032 (2020).

13. Xia, Q.-k., Liu, Z.-y.&Li, Y.-t. Microstructure and properties of Al-Cu-Mg-Ag alloy exposed at 200 °C with and without stress. *Trans. Nonferrous Met. Soc. China* **18**, 789-794 (2008).

14. Fan, J., Yang, B., Wang, Y., Gao, M.&Guan, R. Enhancing the tensile strength and heat resistance induced by high-density Ω phases in an Al–Cu–Mg–Ag alloy. *J. Mater. Res. Technol.* **18**, 3347-3357 (2022).

15. Abdelaziz, M. H., Doty, H. W., Valtierra, S.&Samuel, F. H. Static versus dynamic thermal exposure of transition elements-containing Al-Si-Cu-Mg cast alloy. *Mater. Sci. Eng. A* **739**, 499-512 (2019).

16. Song, M., Xiao, D.&Zhang, F. Effect of Ce on the thermal stability of the Ω phase in an Al-Cu-Mg-Ag alloy. *Rare Met.* **28**, 156-159 (2009).

17. Li, Q., Qin, J., Jiang, D., Yi, D.&Wang, B. Precipitate coarsening and mechanical properties in 6082 aluminium alloy during long-term thermal exposure. *J. Alloy. Compd.* **909**, 164819 (2022).

18. Yuan, W., Liang, Z., Zhang, C.&Wei, L. Effects of La addition on the mechanical properties and thermal-resistant properties of Al–Mg–Si–Zr alloys based on AA 6201. *Mater. Des.* **34**, 788-792 (2012).

19. Chen, B. A. et al. Effect of interfacial solute segregation on ductile fracture of Al–Cu–Sc alloys. *Acta Mater.* **61**, 1676-1690 (2013).

20. Gao, Y. H. et al. Si-mediated reassembly of interfacially segregated Sc atoms in an Al–Cu–Sc alloy exposed to high-temperature creep. *J. Alloy. Compd.* **845**, 156266 (2020).

21. Gao, Y. H. et al. Segregation-sandwiched stable interface suffocates nanoprecipitate coarsening to elevate creep resistance. *Mater. Res. Lett.* **8**, 446-453 (2020).
