# Supplementary material for: Synergy of multiple precipitate/matrix interface structures for a heat resistant high-strength Al alloy
Source: Nat Commun. 2023 May 23;14:2959. doi: 10.1038/s41467-023-38730-z (PMC10205818; doi:10.1038/s41467-023-38730-z)
Supplement: Supplementary file 3 — Description of Additional Supplementary Files [file 41467_2023_38730_MOESM3_ESM.pdf]

Description of Additional Supplementary Files

File Name: Supplementary Movie 1
Description: 3DAP result of the designed alloy in under-aged state. The plate-like C-AlMgSiCu and lath-like L-AlMgSiCu phases are marked in purple color, the χ-AgMg interface phase is marked in green color and the phase in orange color is θ′-Al2Cu. The size of the observation area is about 85 × 85 × 500 nm.

File Name: Supplementary Movie 2
Description: 3DAP result of the designed alloy under peak-aged state. The plate-like C-AlMgSiCu and lath-like L-AlMgSiCu phases are marked in purple color, the χ-AgMg interface phase is marked in green color and the phase in orange color is θ′-Al2Cu. The size of the observation area is about 90 × 90 × 600 nm.

File Name: Supplementary Data 1
Description: Crystallographic information file of C-AlMgSiCu phase reported by Torsæter et al.

File Name: Supplementary Data 2
Description: Crystallographic information file of the newly discovered χ-AgMg interface phase at the θ′/Al interface in this work

File Name: Supplementary Data 3
Description: Crystallographic information file of the uniformly distributed AgMg segregation layer containing double atomic layers at the Ω/Al interface reported by Kang et al.

File Name: Supplementary Data 4
Description: Crystallographic information file of the segregation structure with double Ag atomic layers at the θ′/Al interface reported by Rosalie et al.

File Name: Supplementary Data 5
Description: Crystallographic information file of the ξ-AgMgAl phase reported in this work
